# Implementing Systematic Review at the National Toxicology Program: Status and Next Steps

**DOI:** 10.1289/ehp.1306711

**Published:** 2013-04-01

**Authors:** Linda S. Birnbaum, Kristina A. Thayer, John R. Bucher, Mary S. Wolfe

**Affiliations:** National Institute of Environmental Health Sciences, National Institutes of Health, Department of Health and Human Services, Research Triangle Park, North Carolina, E-mail: bucher@niehs.nih.gov

The National Toxicology Program (NTP), an interagency program headquartered at the National Institute of Environmental Health Sciences (NIEHS), carries out a broad range of toxicology research and testing and serves as a resource for identification of substances in our environment that are hazards for human health. One of the ways that the NTP identifies hazards is through carrying out literature-based health assessments. Approximately 2 years ago we began exploring systematic-review methodology as a means to enhance transparency and increase efficiency in summarizing and synthesizing findings from studies in our literature-based health assessments. A systematic review uses an explicit, prespecified approach to identify, select, assess, and appraise the data from studies that focus on addressing a specific scientific question (Institute of Medicine 2011). Although traditionally used to grade the quality of evidence and strength of scientific support for recommendations for clinical practice guidelines and healthcare interventions [Agency for Healthcare Research and Quality (AHRQ) 2012; Guyatt et al. 2011; Higgins and Green 2011], we—and others—were interested in how systematic review methodology might be applied to environmental health questions (Agency for Toxic Substances and Disease Registry 2012; National Research Council 2011; Silbergeld and Scherer 2013; U.S. Environmental Protection Agency 2013; Woodruff and Sutton 2011).

With the establishment of the Office of Health Assessment and Translation (OHAT) in 2011, the NIEHS launched a new problem-solving resource for the NTP, particularly with respect to identification of noncancer hazards in our environment (Bucher et al. 2011). OHAT took the lead in investigating how systematic review methodology might be used by the NTP. We embraced systematic review methodology as a useful approach for providing thorough documentation of the steps, inputs, and decisions in a literature-based evaluation. However, we also recognized the necessity to extend existing systematic review methods to accommodate our need in environmental health to integrate data from multiple evidence streams (human, animal, *in vitro*) and focus on observational human studies rather than on the randomized clinical trials more commonly encountered in the field of health-care intervention (NTP 2012a, 2012b).

In late February 2013, the NTP released the Draft OHAT *Approach for Systematic Review and Evidence Integration for Literature-based Health Assessments – February 2013* [Draft OHAT Approach; Department of Health and Human Services (DHHS) 2013] for public comment; the deadline for receipt of comments is 11 June 2013. The Draft OHAT Approach adopts or adapts guidance from authoritative systematic review groups (AHRQ 2012; Guyatt et al. 2011; Higgins and Green 2011) to handle the breadth of data from human, animal, *in vitro*, and mechanistic studies relevant for addressing environmental health questions. In developing a draft approach, OHAT sought advice on systematic review through educational webinars and consultation with technical experts, the NTP Executive Committee, a working group of the NTP Board of Scientific Counselors, the NTP Board of Scientific Counselors, and the public. The draft approach involves a seven-step framework for incorporating systematic review methodology into OHAT literature-based health assessments. In early April of 2013, OHAT will release protocols for two case studies to illustrate application of this framework in specific evaluations. We will test our approach in these case studies to help determine whether additional refinement or revision to the Draft OHAT Approach might be needed. To help the public understand the draft approach and protocols, the NTP will hold a web-based informational meeting on 23 April 2013 to provide an overview of the framework, describe the contents of the case-study protocols, and respond to questions (DHHS 2013). Our intent is to carefully consider all public comments received on the draft approach and to present the Draft OHAT Approach to the NTP Board of Scientific Counselors at its meeting on 25–26 June 2013, with discussion by the NTP of any plans to update the document on the basis of the public’s input. Moving forward, our goal is to increase efficiency and provide greater transparency to the rigorous and objective approach that has been the hallmark of OHAT literature-based health assessments.

## References

[r1] Agency for Toxic Substances and Disease Registry (2012). Update on the “Future of Science at ATSDR Symposium.” In: Board of Scientific Counselors Meeting, May 17–18, 2012, Atlanta, Georgia. Atlanta, GA:Agency for Toxic Substances and Disease Registry, 14–17.. http://www.atsdr.cdc.gov/science/docs/BSC%20MINUTES%20MAY%202012.pdf.

[r2] AHRQ (Agency for Healthcare Research and Quality) (2012). Grading the Strength of a Body of Evidence When Assessing Health Care Interventions: An Update (Draft Report).. http://effectivehealthcare.ahrq.gov/search-for-guides-reviews-and-reports/?pageaction=displayproduct&productid=1163.

[r3] Bucher JR, Thayer K, Birnbaum LS (2011). The Office of Health Assessment and Translation: a problem-solving resource for the National Toxicology Program. Environ Health Perspect.

[r4] DHHS (Department of Health and Human Services) (2013). Draft Office of Health Assessment and Translation approach for systematic review and evidence integration for literature-based health assessments—February 2013; request for comments; notice of a meeting. Fed Reg 78(37):12764–12765.. http://www.gpo.gov/fdsys/pkg/FR-2013-02-25/pdf/2013-04254.pdf.

[r5] Guyatt G, Oxman AD, Akl EA, Kunz R, Vist G, Brozek J (2011). GRADE guidelines: 1. Introduction—GRADE evidence profiles and summary of findings tables.. J Clin Epidemiol.

[r6] Higgins JPT, Green S, eds (2011). Cochrane Handbook for Systematic Reviews of Interventions. Version 5.1.0 (updated March 2011).. http://handbook.cochrane.org/.

[r7] Institute of Medicine (2011). Finding What Works in Health Care: Standards for Systematic Reviews. Washington, DC:National Academies Press.. http://www.iom.edu/reports/2011/finding-what-works-in-health-care-standards-for-systematic-reviews.aspx.

[r8] National Research Council (2011). Committee to Review of the Environmental Protection Agency’s Draft IRIS Assessment of Formaldehyde. Washington, DC:National Academies Press.. http://www.nap.edu/openbook.php?record_id=13142.

[r9] NTP (National Toxicology Program) (2012a). National Toxicology Program Board of Scientific Counselors: Summary Minutes, December 11, 2012.. http://ntp.niehs.nih.gov/NTP/About_NTP/BSC/2012/December/BSCMinutes20121211_508.pdf.

[r10] NTP (National Toxicology Program) (2012b). National Toxicology Program Board of Scientific Counselors: Summary Minutes, June 21–22, 2012.. http://ntp.niehs.nih.gov/NTP/About_NTP/BSC/2012/June/BSCMinutes20120622_508.pdf.

[r11] Silbergeld E, Scherer RW (2013). Evidence-based toxicology: strait is the gate, but the road is worth taking.. ALTEX.

[r12] U.S. Environmental Protection Agency (2013). Materials Submitted to the National Research Council. Part I: Status of Implementation of Recommendations.. http://www.epa.gov/iris/pdfs/IRIS%20Program%20Materials%20to%20NRC_Part%201.pdf.

[r13] Woodruff TJ, Sutton P, The Navigation Guide Work Group (2011). An evidence-based medicine methodology to bridge the gap between clinical and environmental health sciences.. Health Aff.

